# Silicon-photonics acoustic detector for optoacoustic micro-tomography

**DOI:** 10.1038/s41467-022-29179-7

**Published:** 2022-03-18

**Authors:** Yoav Hazan, Ahiad Levi, Michael Nagli, Amir Rosenthal

**Affiliations:** Technion - Israel Institute of Technology, Haifa, 3200003 Israel

**Keywords:** Imaging and sensing, Biomedical engineering, Photoacoustics

## Abstract

Medical ultrasound and optoacoustic (photoacoustic) imaging commonly rely on the concepts of beam-forming and tomography for image formation, enabled by piezoelectric array transducers whose element size is comparable to the desired resolution. However, the tomographic measurement of acoustic signals becomes increasingly impractical for resolutions beyond 100 µm due to the reduced efficiency of piezoelectric elements upon miniaturization. For higher resolutions, a microscopy approach is preferred, in which a single focused ultrasound transducer images the object point-by-point, but the bulky apparatus and long acquisition time of this approach limit clinical applications. In this work, we demonstrate a miniaturized acoustic detector capable of tomographic imaging with spread functions whose width is below 20 µm. The detector is based on an optical resonator fabricated in a silicon-photonics platform coated by a sensitivity-enhancing elastomer, which also effectively eliminates the parasitic effect of surface acoustic waves. The detector is demonstrated in vivo in high-resolution optoacoustic tomography.

## Abstract (web summary)

High-resolution photoacoustic tomography is challenging due to reduced efficiency of miniaturized piezoelectric elements. Here, the authors fabricate a miniaturized silicon-photonics acoustic detector coated with a sensitivity-enhancing elastomer, and demonstrate high-resolution in vivo imaging.

## Introduction

Multi-element piezoelectric transducers have been one of the enabling factors for clinical applications in the field of ultrasound and optoacoustic imaging thanks to their ability to simultaneously generate and detect acoustic signals. However, array-based configurations are commonly designed for sub-millimeter resolutions, whereas better resolutions require bandwidths and miniaturization levels that are often impractical for piezoelectric technology. Generally, tomographic configurations require that the detector size be comparable to the smallest feature size in the image, but the miniaturization of piezoelectric detectors comes at the expense of sensitivity. In the field of optoacoustic tomography (OAT), which is characterized by lower signal levels than those found in medical ultrasound^1^, bulky detectors are often required to detect the minute acoustic signals, limiting the achievable resolution. As a result, high-resolution modalities such as acoustic microscopy^2^ and acoustic-resolution photoacoustic microscopy (AR-PAM)^3–6^ are not based on tomographic configurations with miniaturized elements, but rather on large ultrasound transducers that are focused to small regions (<50 µm).

**Fig. 1:**
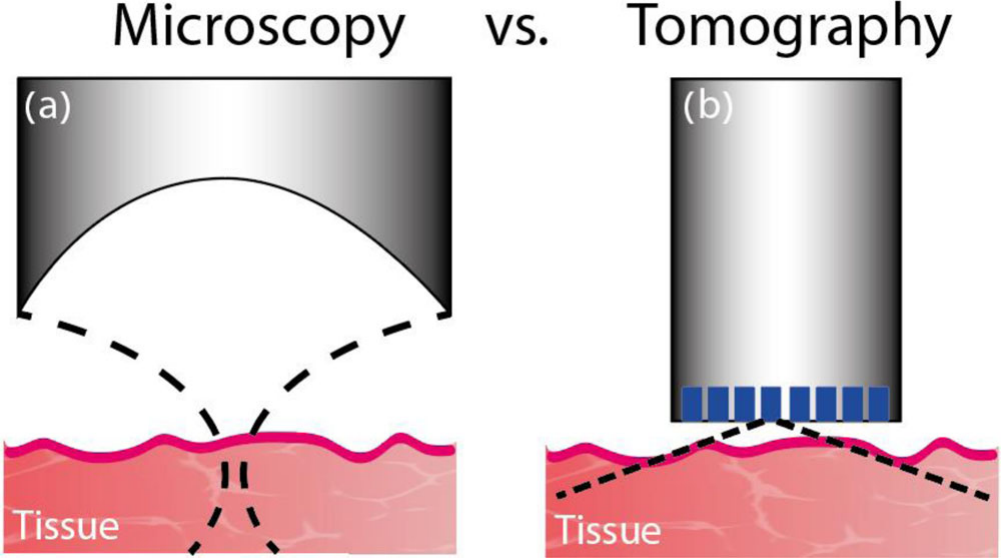
Detection schemes in optoacoustic (photoacoustic) imaging. **a** Microscopy: a large detection surface is focused on a small region in the tissue to achieve high lateral resolution. Mechanical scanning is essential for image formation. High lateral resolution requires strong acoustic focusing, i.e., a high numerical aperture, leading to short depths of field. **b** tomography: a detector whose size is comparable to the acoustic wavelength can detect ultrasound over a wide angular coverage. The acoustic measurement is performed for numerous positions over the detection surface, either via mechanical scanning or stationary arrays, and a tomographic reconstruction algorithm is used to form the image. There is no inherent limitation on the imaging depth, but acoustic attenuation and the limited scanning span pose practical limitations on the resolution that may be achieved for a given depth^11^.

Figure 1 shows a comparison between the detection geometries of AR-PAM and OAT, elucidating the fundamental difference between the two approaches. In AR-PAM, the large detector size, required for strong focusing, enables high sensitivity but inherently limits the production of dense detector arrays, which are common in OAT. In addition, the optimal performance of AR-PAM is obtained only in its focal region, leading to a tradeoff between its resolution, determined by the width of the focal region, and depth of field^7^. Conventionally, AR-PAM systems operate with a typical frequency of 50 MHz and a focal spot of 50 µm and depth-of-field of 300 µm, but high-resolution implementations of AR-PAM, e.g., raster-scanning optoacoustic mesoscopy^4,8^ (RSOM), have been demonstrated with central frequencies up to 100 MHz and focal widths of 20 µm, resulting in depths of fields of 100 µm and lower. While imaging beyond the depth of field is possible using synthetic-aperture approaches^9^, the resulting reconstructions may suffer from lower sensitivities or resolutions than in the focus^10^. In the case of RSOM, the extremely short depth of fields necessitates imaging outside the focus. Even when the depth of field is sufficient for a given application, to achieve optimal performance AR-PAM still requires that the imaged region is at the correct distance from the transducer, complicating its use when the surface of the imaged organ is not parallel to the detection surface. A summary of the common AR-PAM implementations is given in Supplementary Note 2.

In contrast to AR-PAM and RSOM, in OAT the detector would ideally have an isotropic sensitivity that is free from the geometrically imposed depth-of-field limitation of focused detectors. Accordingly, OAT can image 3D volumes without the need to vertically scan the detector and achieve resolutions and penetration depths that are ultimately limited by optical and acoustic attenuation in biological tissue^11^. However, to achieve close to isotropic sensitivity, the detector’s lateral dimensions need to be miniaturized to the level of half the acoustic wavelength^12^. In practice, most ultrasound detectors do not have an isotropic angular sensitivity, which can potentially deteriorate the lateral resolution of the OAT image^13^. Although the lateral resolution is generally comparable to the detector width in the case of a planar detection surface^7^, its exact value depends on the detector’s shape, bandwidth, and type of signal processing performed, as discussed in Supplementary Note 1.

Optical techniques enable the high-efficiency generation and detection of wideband (>100 MHz) ultrasound with elements on the scale of 10 µm^1,14–16^, potentially facilitating the development of micro-tomography systems that achieve microscopic resolutions using tomographic configurations. One of the most common techniques for optical detection of ultrasound is based on monitoring the wavelength modulation of optical resonators due to ultrasound-induced deformations and refractive-index perturbations^1^. Often, polymer resonators are preferred because they exhibit a high elasto-optic response, which leads to high sensitivity, and an acoustic impedance that is relatively matched to that of biological tissue and water, reducing the parasitic effect of acoustic reverberation and surface acoustic waves (SAWs), which hinders signal fidelity. Despite their excellent acoustic properties, polymer resonators are not ideal candidates for fabricating miniaturized ultrasound detectors since their low refractive-index contrast leads to relatively poor confinement of the optical mode, limiting the achievable Q-factor, sensitivity, and size^8^.

Silicon photonics represent a promising alternative to polymer resonators, which can achieve an unparalleled combination of miniaturization and sensitivity^17–20^, while offering the advantages of a low-cost fabrication via CMOS-compatible processes. Recently, the facet of a silicon waveguide was demonstrated for ultrasound detection with a noise equivalent pressure (NEP) of 9 mPa Hz^−1/2^ and bandwidths reaching 230 MHz with sub-micron detector size^18^. Using this detector, high image resolution and contrast were achieved in a near-field optoacoustic measurement. However, near-field imaging, performed at distances of 10 s of microns from the detector, is incompatible with most in vivo applications of optoacoustics. More recently, far-field OAT of simple objects has been demonstrated using an acoustic membrane fabricated over a silicon-photonics microring^19^, which achieved NEPs of 1.3 and 2.3 mPa Hz^−1/2^ with membrane diameters of 20 and 15 µm, respectively. However, acoustic membranes are inherently resonant structures, which limited the achieved bandwidths to 27 MHz^19^. A major challenge in using silicon-photonics detectors for OAT is the high acoustic impedance of silicon, making it susceptible to acoustic reverberations and SAWs, which severely limit the accuracy of the tomographic reconstruction. Although the high-signal fidelity of polymer resonators has enabled the generation of in vivo OAT images with exceptional quality^21^, silicon-photonics-based OAT has been generally restricted to simple objects and has not been demonstrated in vivo.

In this work, we report on a miniaturized silicon-photonics acoustic detector (SPADE) and demonstrate its capability for in vivo optoacoustic micro-tomography. Our platform is based on a π-phase-shifted Bragg grating (π-BG) resonator in a silicon waveguide coated with the elastomer polydimethylsiloxane (PDMS) combined with a low-noise interferometric setup for signal readout. The PDMS coating enhances the sensitivity and reduces the parasitic effect of SAWs, which has limited the imaging capability of previous silicon-photonics detectors^17,22^. SPADE is demonstrated with NEPs down to 2.2 mPa Hz^−1/2^ and a bandwidth above 200 MHz, corresponding to a theoretically achievable axial resolution of ~6 µm. The performance of SPADE for optoacoustic micro-tomography was tested for point sources, revealing lateral and axial spread functions with widths below 20 µm and a typical two-point resolution^23^ of 25 µm. When tested on a resolution target, our imaging system successfully resolved structures with up to 22.6 lines per millimeter. The imaging resolution achieved by SPADE, which is comparable to the most advanced implementations of AR-PAM (Supplementary Note 2), is unparalleled in the field of OAT and is the result of not only its advanced technical characteristics but also its readily decipherable signals, relatively free of parasitic effects and governed mainly by the time-of-flight principle. A comparison between the performances of SPADE to leading optical techniques for ultrasound detection is provided in Supplementary Note 3.

## Results

### SPADE system setup

**Fig. 2:**
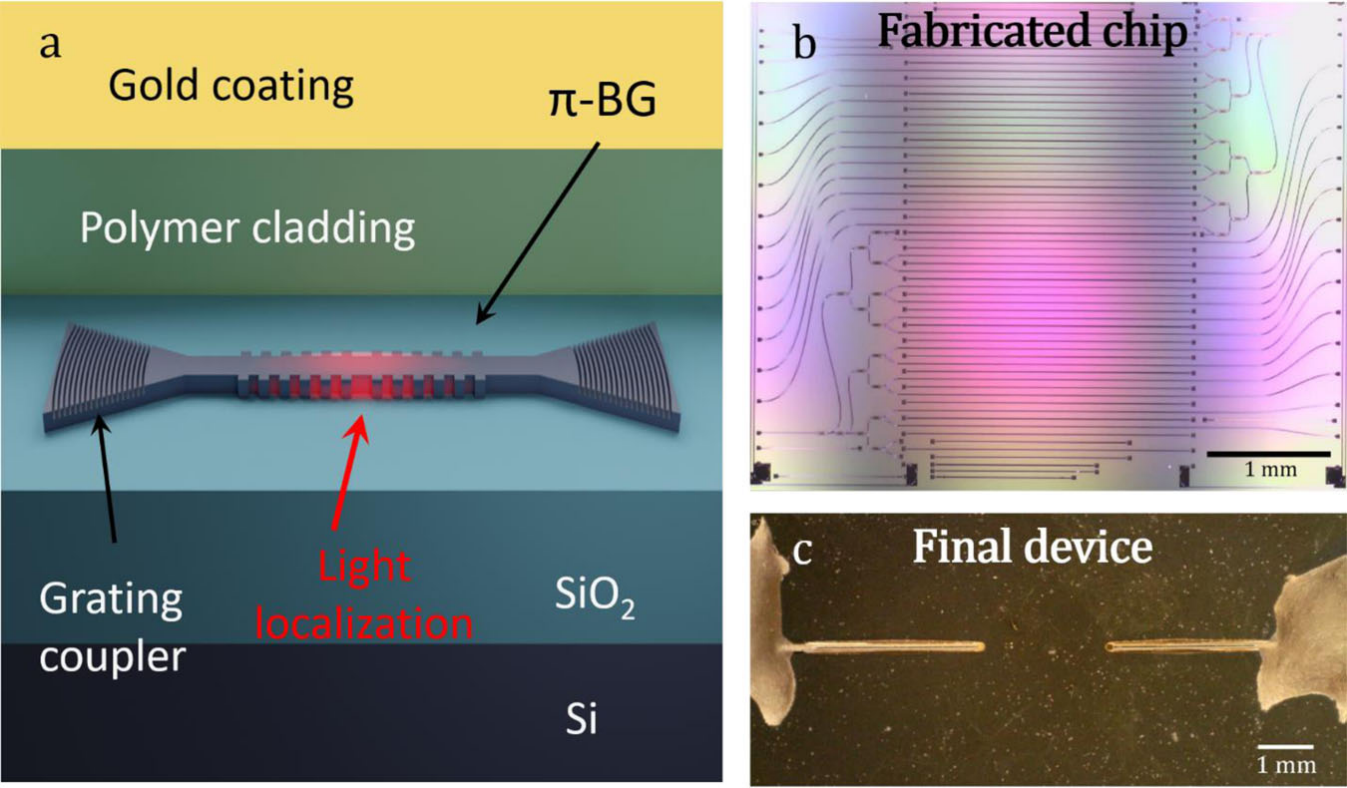
SPADE: Silicon-photonics acoustic detector. **a** Illustration of the silicon-photonics layer structure and the structure of π-phase-shifted Bragg grating (π-BG) with grating couplers. **b** Photograph of the silicon-photonics chip prior to fiber bonding. **c** Photograph of the assembled and gold-coated silicon-photonics chip.

An illustration of the sensing element in SPADE is shown in Fig. 2a. The π-BG is composed of two side-corrugation Bragg reflectors separated by a $\lambda /4$ spacer, creating a localized cavity mode. Since acoustic sensing is performed only in the illuminated regions of the resonator^24^, localization of the cavity mode enables miniaturization of SPADE below the physical length of the π-BG. Light is coupled to the π-BG via vertical grating couplers and angle-polished polarization-maintaining optical fibers^22^. The silicon core coated with a PDMS over-cladding and a top gold layer deposited to reflect stray light from the optoacoustic illumination, thus reducing the temperature increase in the chip due to the absorption of the stray pulse light by the silicon substrate. Figure 2b, c, respectively, shows photos of the entire silicon chip after fabrication and the final device after fiber coupling and gold coating. The silicon chip included numerous waveguides with different π-BG designs, but fiber coupling was performed only to one of the waveguides. Although the individual waveguides are clearly seen in the microscope image, the structure of the nanometer-scale structure of the π-BGs cannot be resolved using optical microscopy.

**Fig. 3:**
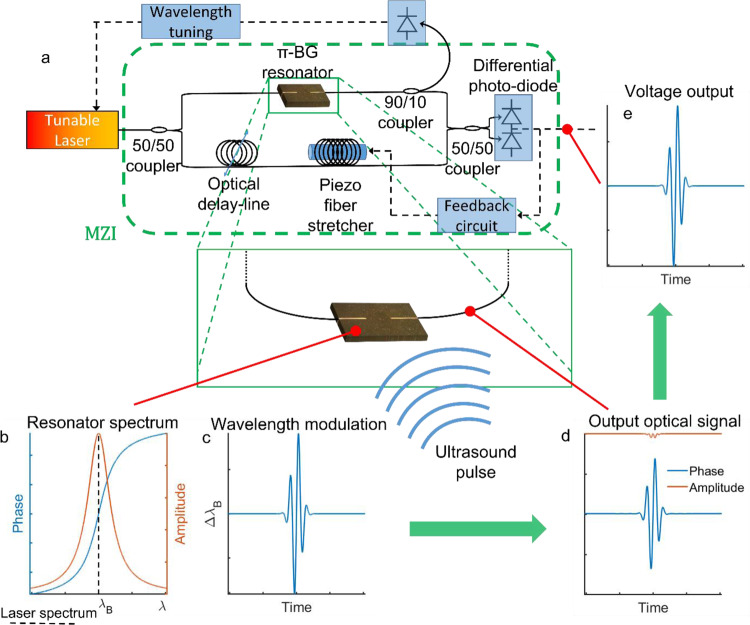
Optical interrogation and readout system. **a** Illustration of the optical readout system. The plots show the resonator’s spectrum (**b**), ultrasound-induced wavelength modulation (**c**), and the corresponding optical output of the resonator (**d**). **e** The corresponding differential voltage signal, sampled at the output of the system. π-BG is π-phase-shifted Bragg grating.

The optical setup used for reading out the signal from the π-BG resonator is shown in Fig. 3. Since the central wavelength of the π-BG, ${\lambda }_{B},$ is pressure-sensitive, when an acoustic wave impinges on the resonator it leads to a modulation in ${\lambda }_{B}$^25^. A continuous-wave (CW) laser is tuned to the transmission peak of the resonance, where the phase response is linear, leading to phase modulation at the output of the resonator that is proportional to the modulation in ${\lambda }_{B}$. To detect the phase modulation, the resonator output interferes with a reference beam in a Mach-Zehnder interferometer (MZI) stabilized to quadrature, leading to a differential output voltage signal that is proportional to the phase modulation^26^. This scheme involves two mechanisms for sensitivity enhancement. First, the optical path difference between the two arms of the MZI is set to approximately zero to enable the cancellation of phase noise from the laser. Second, interfering with the signal with a high-power reference beam effectively amplifies the signal, partially compensating for coupling losses. We note that the scheme of Fig. 3 is generic and it may be applied to improve the sensitivity of other resonator-based detectors.

### SPADE performance

The π-BG was fabricated and characterized for both the transverse-electric (TE) and transverse-magnetic (TM) modes. For both polarizations, the cross-sectional dimensions of the modes were smaller than 1 µm, whereas the effective longitudinal dimensions were ~30 µm and 200 µm for the TE and TM resonators, respectively. The longitudinal dimensions are a result of light localization, leading to an exponentially decaying sensitivity profile from the π-phase shift^24^. As the coupling between the forward and backward-propagating modes in the π-BG was weaker in the TM mode, leading to a narrower bandgap (Supp. Fig. 2), its light localization was lower, leading to an effectively longer sensor. Accordingly, the TE mode is more appropriate for 3D OAT, in which high resolution is desired in both lateral dimensions (Supplementary Note 1), whereas the TM mode could be useful in 2D cross-sectional OAT or ultrasound^27^, in which high resolution is obtained only in plane.

**Fig. 4:**
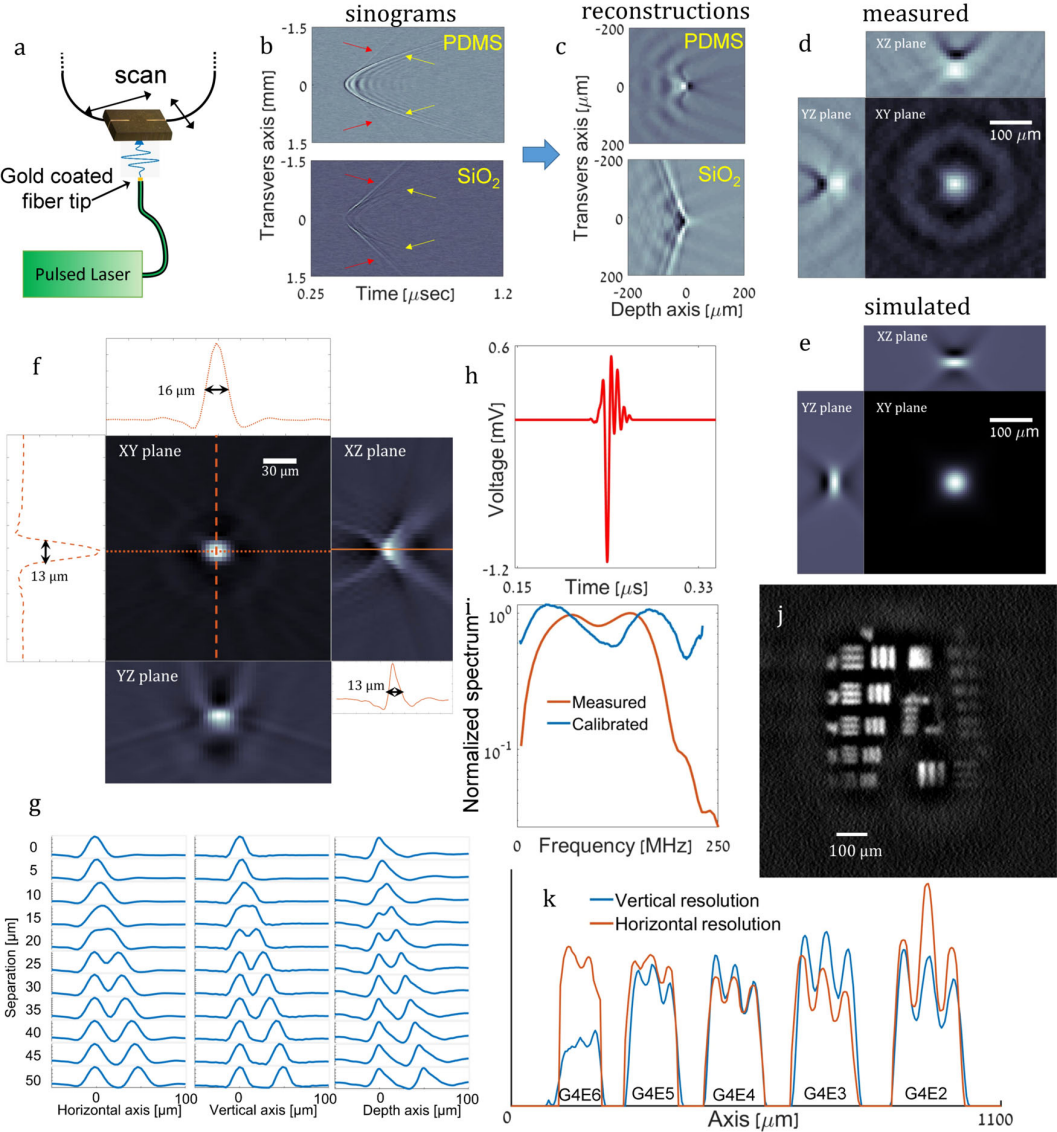
Performance of SPADE. **a** Schematics of the experimental system in which an optoacoustic point source was created by illuminating the gold-coated tip of an optical fiber with optical pulses. **b** Sinogram measurements were obtained with SPADE with PDMS over-cladding (top) and Si0_2_ over-cladding (bottom), where the coated-fiber source had a core diameter of 50 µm. Red arrows mark surface acoustic waves, yellow arrows mark direct-impact acoustic waves. **c** Corresponding 2D optoacoustic reconstructions obtained from the sinograms of **b**. **d** 3D Reconstruction cross-sections obtained for the measurement and simulation (**e**) of the 50 µm source. **f** Reconstruction cross-section of the 10 µm source and corresponding 1D slices, revealing reconstruction transversal widths of 13 µm and 16 µm, and depth of 13 µm. **g** Separation simulation of two 10 µm sources at different distances for horizontal, vertical, and depth shifts. **h** Acoustic signal measured by the PDMS-coated SPADE for a coated-fiber source with a core diameter of 10 µm. **i** The spectrum of the acoustic signal presented in **h** (red) and the calibrated spectrum. **j** Reconstruction of resolution target 1951 USAF groups 4–7. **k** one-dimensional cross-sections of resolution target, G marks group number, and E marks the element number.

To characterize the acoustic response of the detectors, a point-like optoacoustic source was generated using an optical fiber with a gold-coated tip, coupled to high-power laser pulses with a duration of 1 ns (Fig. 4a). In the first set of measurements, the fiber core was 50 µm—sufficiently large to generate acoustic signals detectable by a calibrated hydrophone. By comparing the signals measured by SPADE to the one measured by the hydrophone for the same distance, NEPs of 9.8 mPa Hz^−1/2^ and 2.2 mPa Hz^−1/2^ were calculated for the TE and TM modes, respectively. By scanning the detector in plane (Fig. 4a), we characterized the spatial response of SPADE and compared it to the response achieved when a silica over-cladding is used, rather than PDMS. Figure 4b shows the sinograms obtained for a 1D scan of two TE resonators, one coated with silica and the other with PDMS. For the PDMS case, the dominant signal in the sinogram had a hyperbolic shape that followed the time-of-flight principle: the signal delay was equal to the distance of the source from the detector divided by the speed of sound. In contrast, the dominant signal of the silica-coated resonator exhibits a linear behavior in its sinogram, expected for SAWs^17^. Figure 4c shows the 2D back-projection reconstructions obtained from the two sinograms of Fig. 4b. Clearly, although the PDMS-coated resonator obtained a point-like image, theoretically expected for such a scan, the reconstruction of the silica-coated resonator suffered from severe distortions and artefacts that limit imaging of complex objects. Figure 4d shows the amplitude cross-sections of the full 3D reconstruction obtained for a 2D scan compared with the theoretical reconstruction obtained with an ideal point detector in Fig. 4e, demonstrating the high imaging fidelity of SPADE.

To test the full capabilities of SPADE in terms of resolution, we repeated the characterization measurement but using an optical fiber with a core of 10 µm and only the PDMS-coated TE resonator. The acoustic signal, measured at a distance of 0.35 mm, and its corresponding spectrum are shown in Fig. 4h, i, respectively. We note that the measured spectrum represents the product of the inherent frequency response of SPADE, the source spectrum, and the electric response of the photodetectors. Calibrating the measured spectrum by the theoretically expected response of an optoacoustic point source and by the measured response of the photodetector, the intrinsic frequency response of SPADE is obtained (blue curve in Fig. 4i), demonstrating a bandwidth beyond 200 MHz, which corresponds to a theoretical axial spread function with a width of 6 µm.

In order to demonstrate the capability of SPADE for performing high-resolution OAT, the optoacoustic source was scanned in 2D and the measured acoustic signals were used to tomographically form an image. The image cross-sections are shown in Fig. 4f, demonstrating reconstruction widths of 16 µm and 13 µm in the lateral dimensions, and 13 µm in the axial direction. To assess the two-point resolution of our system, i.e., its ability to resolve two-point sources in close proximity^23^, we replicated the reconstruction of Fig. 4f and added it to the original reconstruction with a shift in either the horizontal, vertical, or depth axis. The 1D slices of the resulting two-point reconstructions are shown in Fig. 4g for different separation values, demonstrating a typical resolution of 25 µm for all axes. To further demonstrate the ability of the system to resolve small structures, Group 4 of a 1951 USAF resolution target was imaged (Fig. 4j), demonstrating a resolution of 22.6 lines per millimeter (Fig. 4j: Group 4, Element 4). In comparison, AR-PAM operating with a central frequency of 50 MHz^28^ has been shown to achieve a resolution of 10.1 lines per millimeter (Group 3, Element 3).

**Fig. 5:**
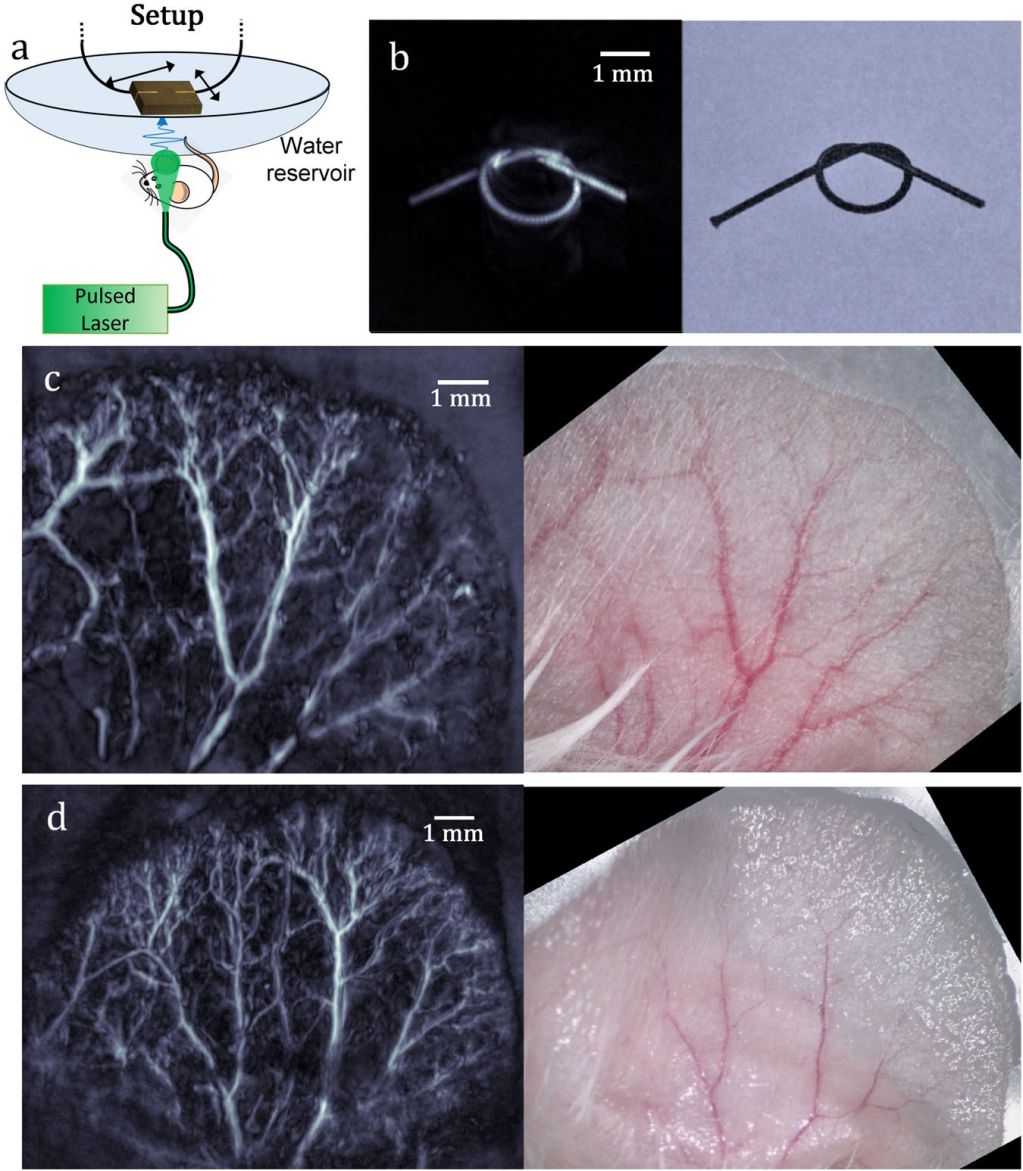
Tomographic imaging via SPADE. **a** Schematics of the experimental system. **b** Maximum intensity projection of the optoacoustic image (left) of a suture and corresponding microscope image (right). **c**, **d** Maximum intensity projections of optoacoustic image (left) of two mouse ears and corresponding microscope images (right).

The ability of SPADE to perform high-fidelity OAT of complex structures was demonstrated both ex vivo and in vivo. In the ex vivo measurement, the imaged object was a dark suture tied into a knot, while in the in vivo measurement, the vasculature of a mouse ear was imaged. In both cases, the imaged object was illuminated from the bottom with 7 ns pulses at the wavelength of 532 nm and the TE resonator was scanned in 2D over a plane above the object to collect the tomographic data used to form the optoacoustic images (Fig. 5a). Figure 5b shows the maximum amplitude projection of the suture reconstruction, whereas Fig. 5c, d shows the maximum amplitude projections for two mouse ears; in all cases, the optoacoustic images are compared with the optical images. The smallest blood vessel that could be resolved in the reconstruction had a width of ~35 µm in both lateral and axial axis. We note that the higher lateral width, in comparison to the measurements of Fig. 4, was expected due to the longer pulse duration used for the optoacoustic excitation^8^. Nonetheless, the lateral widths demonstrated in the in vivo measurements are typical for AR-PAM systems operating at a central acoustic frequency of 50 MHz^28^.

## Discussion

SPADE enables high-fidelity OAT at resolutions that have been previously attainable only by microscopy schemes. Since micron-scale light localization in silicon photonics is possible, the lateral resolution of SPADE may be further improved beyond the values obtained in this work. In the case of axial resolution, the theoretical value of 6 µm merely represents the limitation theoretically calculated from the bandwidth that could be measured in our setup. By using photodetectors with higher bandwidths, higher axial resolutions may be achieved with values comparable to the sub-micron height of the silicon waveguide. However, such axial resolutions, corresponding to a bandwidth of up to 5 GHz (Supplementary Note 1), would involve extremely high acoustic losses in the tissue, and possibly in the over-cladding coating material, severely limiting the imaging depth.

SPADE-based optoacoustic micro-tomography offers two potential advantages over AR-PAM systems. First, a tomographic geometry enables the reconstruction of large 3D volumes from 2D scans, whereas the use of focused detectors in AR-PAM leads to a stringent limitation on the depth of field. Second, in contrast to AR-PAM, which is geometrically limited to a single large-area detector, the configuration of SPADE may be scaled up to dense arrays to increase the imaging rate and potentially enable scan-free tomographic imaging. Such a scale-up would be facilitated by the proven capabilities of silicon photonics for array fabrication^19,29^ and recent developments in parallel signal readout from optical resonators^30–32^ using wideband interrogation sources. Specifically, arrays may be produced with separations smaller than 10 µm without any cross-talk^33^, facilitating the development of dense arrays.

Considering that the width of the sensing elements is smaller than 1 µm and that the ideal spacing between the elements is half the acoustic wavelength, on the scale of 10 µm for the frequencies studied in this work, a full 1D array with 64–128 elements could be fabricated in a 1 mm device, potentially enabling scan-free cross-sectional imaging and even 3D imaging when compressed-sensing or deep-learning reconstruction algorithms are used^34,35^. This level of miniaturization can enable integrating SPADE’s imaging capabilities in endoscopes and laparoscopes used in minimally invasive procedures, leading to new clinical applications. Clearly, since parallel signal readout is generally associated with lower sensitivities^30^, such SPADE arrays will involve a tradeoff between penetration and imaging rate. Nonetheless, the expected loss of sensitivity due to parallelization may be mitigated by new strategies to increase the acoustic response of SPADE. Specifically, SPADE’s sensitivity may be improved by using low-loss platforms, in which higher Q-factors may be achieved^36,37^, and by using over-cladding materials with a higher elasto-optic response.

## Methods

### SPADE fabrication

The π-BGs were fabricated using CMOS technology at IMEC in two versions: with silica over-cladding deposition and with a resist polymer applied directly on the Si layer. The chip with the resist polymer was further processed: the resist was chemically removed with acetone and then spin-coated with PDMS polymer (LS-6941) layer of ~2 µm as an over-cladding layer. The PDMS underwent heat curing to reach its final mechanical properties of viscosity and elasto-optic coefficients. For both versions of π-BGs, two polarization-maintaining fibers polished at 42.5° were bonded with refractive-index-matching glue (THORLABS, NOA61), coupling light in and out of the silicon waveguide through grating couplers. This fiber-to-chip coupling configuration results in a planar structure, not limiting the distance from the source to the sensor by more than the fiber diameter. Finally, a 200 nm layer of gold is deposited on the chip and the bonded fibers.

The π-BG design was strongly polarization-dependent since the modal cross-section of the TE and TM polarization differ greatly (Supplementary Fig. 2g, h). Therefore, the π-BG design for each polarization differs significantly in its geometrical dimensions. The TE π-BG dimensions were: 265 µm long, 500 nm wide with 40 nm corrugation depth, and 225 nm high. The TM π-BG dimensions were: 1537 µm long, 500 nm wide with 100 nm corrugation depth, and 225 nm high. The transmission spectra of both π-BG designs are presented in Supplementary Fig. 2i, j.

### Elasto-optic properties of the PDMS over-cladding

When a resonator is used to detect ultrasound, its response may be quantified by the normalized sensitivity function^38^ ${S}_{\lambda }=d{\lambda }_{{{{{\rm{res}}}}}}/({\lambda }_{{{{{\rm{res}}}}}}dP)$, where ${\lambda }_{{{{{\rm{res}}}}}}$ is the resonance wavelength, $d{\lambda }_{{{{{\rm{res}}}}}}$ is the shift in resonance wavelength due to the acoustic perturbation $dP$. The ultrasound-induced resonance wavelength shift is a result of two mechanisms: effective-refractive-index modulation due to the elasto-optic effect and the geometrical modulation of the resonator’s structure. The relation of two mechanisms to the normalized sensitivity is given by ${S}_{\lambda }=d{n}_{{{{{\rm{eff}}}}}}/({n}_{{{{{\rm{eff}}}}}}dP)+d{\varepsilon }_{z}/dP$, where ${n}_{{{{{\rm{eff}}}}}}$ is the effective-refractive index of the guided mode, and ${\varepsilon }_{z}$ is the strain in the z direction. Generally, $d{n}_{{{{{\rm{eff}}}}}}$ is determined by both the change in the refractive index of the materials composing the waveguide due to the elasto-optic effect and changes in the core geometry. However, for cladding materials with the sufficiently high elasto-optic response, geometrical effects may be neglected, leading to^39^ $\varDelta n{}_{{{{{\rm{eff}}}}}}\propto {\iint }_{{{{{\rm{PDMS}}}}}}\varDelta n(x,y){|e(x,y)|}^{2}dxdy$, where ${|e(x,y)|}^{2}$ is the normalized mode energy and $\varDelta n$ is the refractive-index perturbation in the cladding, given by:^22^

1a $$\varDelta {n}_{x}=\frac{({C}_{1}\nu +{C}_{2})}{1-\nu }{\sigma }_{y}$$

1b $$\varDelta {n}_{y}=\frac{[(1-\nu ){C}_{1}+2\nu {C}_{2}]}{1-\nu }{\sigma }_{y}$$

where ${\sigma }_{y}$ is the acoustically induced stress in the $y$ direction (perpendicular to the SPADE plane), ${C}_{1}$ and ${C}_{2}$ are the elasto-optic constants, and $\nu$ is the Poisson ratio. Although we are not aware of the exact values of ${C}_{1}$ and ${C}_{2}$ for PDMS, birefringence measurements performed in transparent rubbers^40^ generally indicate values that are above 10^3^ TPa^−1^, i.e., over an order of magnitude higher than the values reported for BCB, previously used as a sensitivity-enhancing coating^20,25^.

A second important acousto-elastic property of PDMS exploited in SPADE is its very high acoustic losses due to its high viscosity, which have shown significant signal attenuation for propagation length on the scale of 10 µm for acoustic frequencies of 10 s of MHz^16^. These high acoustic losses are negligible for direct ultrasound waves that propagate through ~2 µm of PDMS, but are significant for SAWs, which propagate 100 s of micrometers in the case of silica-coated resonators, as can be seen in Fig. 4b.

### Optical interrogation system

A tunable CW laser (Santec, TSL-550) connected to a balanced, polarization-maintaining fiber-based MZI. One arm of the MZI was connected to an optical delay-line (OZ optics, ODL-100) and a piezoelectric fiber stretcher (OPTIPHASE, PZ3) with a feedback circuit with a bandwidth of 3 kHz; the second arm was connected to the SPADE. From the output of the SPADE, 10% of the power was sampled by a photodetector (THORLABS, PDA50B-EC). The photodetector signal is used to lock the tunable laser’s wavelength to the maximum transmission of the π-BG. The difference between the two outputs of the MZI is measured by a differential photodetector with an electrical bandwidth of 150 MHz. The differential photodetector voltage signal was split into two outputs: one used for the feedback signal that locked the MZI to quadrature and the other sampled by an analog to digital converter (M3i.4860-Exp, SPECTRUM). The voltage output of the interrogation system is given by^26^

2 $$V(t)={V}_{0}\sqrt{T}[{\phi }_{{{{{\rm{US}}}}}}(t)+{\varphi }_{n}(t-\varDelta {T}_{1})-{\varphi }_{n}(t-\varDelta {T}_{2})]$$

where ${V}_{0}$ is a scaling constant, $T$ is the SPADE power transmission, ${\phi }_{{{{{\rm{US}}}}}}(t)$ is the ultrasound-induced modulation of the phase at the output of the resonator, and ${\varphi }_{n}$ is the laser phase noise. The advantages of our phase-detection approach over conventional intensity detection can be understood from the equation above. First, signal reduction due to coupling losses to the SPADE chip is mitigated by the interference with the reference beam, leading to an output signal that is proportional to $\sqrt{T}$, and not $T$. With coupling losses as high as −16 dB in our experiments, this corresponds to sixfold gain in voltage signal. Second, by balancing the optical path length in both arms to achieve $\Delta {T}_{1}=\Delta {T}_{2}$, laser phase noise, which is the main noise source in many intensity-based interrogation schemes^41^, is effectively eliminated.

### Fiber optoacoustic source fabrication and setup

A cleaved optical fiber was coated with a 200 nm layer of gold using vapor deposition. The gold layer acted as an optoacoustic source of the same size as the fiber core (50 µm and 10 µm cores were used in this work). The gold-coated tip was submerged in a water bath along with the SPADE, which was scanned in the proximity of the fiber tip. The light was coupled to the fiber through its uncoated distal side from an Nd:YAG pulse laser with a pulse energy of 120 µJ, pulse duration of 1 ns, a wavelength of 1064 nm, and repetition rate of 1 kHz (OPTOGAMA, WAVEGUARD).

### Image reconstruction

The reconstruction of the optoacoustic images from the raw signals was performed by MATLAB in two steps. In the first step, the raw data was spatially deconvolved with the function that describes the effective sensing region of the detector to improve the lateral resolution, as discussed in Supplementary Note 1. Since the width of the TE mode sensor is <1 µm, whereas its length sensitivity is theoretically expected to have an exponential profile with a width of ~30 μm^17^, the deconvolution was performed only in one longitudinal dimension of the 3D data set. In the second step, the imaged was formed from the spatially deconvolved signals using the universal back-projection algorithm^42^.

### In vivo imaging

A laboratory mouse model (ICR(CD-1), female, age 14–16 weeks) was anesthetized using isoflurane and placed under an IR heating lamp to maintain its body temperature. The mouse ear was illuminated from the bottom with optical pulses (INNOLAS, SpitLight EVO OPO) spatially diffused to uniformly illuminate the entire ear. The illumination had the following parameters: a wavelength of 532 nm, pulse duration of 7 ns, and pulse energy (after the diffuser) of 300 µJ. The top side of the ear was acoustically coupled using a drop of water to a water reservoir bounded by a thin polyethylene membrane. The SPADE was submerged in the water reservoir and scanned over the mouse’s ear at a distance of 5 mm, a span of 15 mm × 15 mm, and 30 µm step size.

### Reporting summary

Further information on research design is available in the Nature Research Reporting Summary linked to this article.

## Supplementary information

Supplementary Information

Reporting Summary

## Data Availability

The data sets generated during and/or analyzed during the current study are available from the corresponding author on request.

## Code availability

The codes used during the current study are available from the corresponding author on request.
