# Supplementary material for: Silicon-photonics acoustic detector for optoacoustic micro-tomography
Source: Nat Commun. 2022 Mar 18;13:1488. doi: 10.1038/s41467-022-29179-7 (PMC8933411; doi:10.1038/s41467-022-29179-7)
Supplement: Supplementary file 1 — Supplementary Information [file 41467_2022_29179_MOESM1_ESM.pdf]

Silicon-photonics acoustic detector for opto-acoustic micro-tomography

Supplementary material

Yoav Hazan, Ahiad Levi, Michael Nagli and Amir Rosenthal

Supplementary Note 1: Theoretical spread function in OAT.

For a given planar scanning geometry, the widths of the axial and lateral spread functions, Δw_a and Δw_l, may be estimated from the bandwidth, Δf, and lateral dimension of the detector, D, of the detector using the following equations[1]:

$$\Delta w_a = \frac{0.8v}{\Delta f} \tag{1a}$$

$$\Delta w_l = \sqrt{D^2 + \Delta w_a^2} \tag{1b}$$

where v is the speed of sound in the medium, which is approximately equal to 1500 m/s in water and tissue. Supplementary Equations 1a,b show that the resolution in the axial direction is exclusively determined by the bandwidth of the detector, whereas the lateral resolution by both the bandwidth and lateral dimensions of the detector. Using the parameters of SPADE, a detector with a bandwidth of 230 MHz and lateral dimension of 0.5 µm and 30 µm, would result in $\Delta w_a = 5.2$ µm, $\Delta w_{l,1} = 5.2$ µm, and $\Delta w_{l,2} = 30.5$ µm.

Supplementary Equations 1a,b are based on the analysis of Xu *et al.*[2] in which the detector was a disc with a diameter D with uniform sensitivity, and the lateral spread function was obtained by convolving the image with the sensitivity distribution of the detector. Since the sensitivity distribution of SPADE is not uniform, but rather exponentially decaying from its center[3], the resulting lateral resolution may be different than in the case of a uniform disk detector. Moreover, spatially deconvolving the acoustic data over the direction of $\Delta w_{l,2}$ can effectively reduce the broadening. Although deconvolution is generally associated with amplification of noise at high spatial frequencies, where the response of the detector is low, this effect is mitigated by the exponentially decaying sensitivity profile of SPADE, whose Fourier transform, a Lorentzian function, is a slowly decaying function in high spatial frequencies. In contrast, Gaussian sensitivity profiles, which are common in Fabry-Perot resonators[4] are only marginally compatible with deconvolution because their Fourier transform decays rapidly at high spatial frequencies.

The ultimate limitation on the detector bandwidth is determined by the height of the sensing zone, since the acoustic wave is convolved with the volume of the detector, rather than just in the 2 lateral directions. Accordingly, acoustic waves that vertically impinge on the detector will be averaged out over the sensing volume if their wavelength is equal to the height of the detector. This effect leads to the following estimate for the detector bandwidth:

$$\Delta f = \frac{v}{2h} \tag{2}$$

where h is the height of the detector. Assuming a speed of sound of 1000 m/s for PDMS and height of 200 nm, representative of the optical mode (Supplementary Fig. 2g,h), a bandwidth of 2.5 GHz is obtained.

Supplementary Note 2: Comparison to acoustic-resolution photoacoustic microscopy.

Optoacoustic tomography requires that the acoustic sensors have a size comparable to the imaged features. However, since the sensitivity of piezoelectric ultrasound transducers scales with size, their miniaturization to levels below 100 µm becomes impractical. Acoustic-resolution photoacoustic microscopy (AR-PAM) overcomes this limitation by using a focused ultrasound transducer, which receives signals from a small region (Fig. 1a), rather than from the entire imaged volume, as in optoacoustic tomography (Fig. 1b). Since the aperture size of the focused transducer is considerably larger than width of the focal regions, high sensitivity and resolution may be achieved together.

While the exact performance characteristics of a given AR-PAM depend on the specific transducer parameters, it may be generally divided into two categories: conventional AR-PAM, operating at a central frequency of 50 MHz[5], and raster-scan optoacoustic mesoscopy (RSOM)[6], operating at a central frequency of approximately 100 MHz[7]. Supplementary Table 1 contains a comparison between these two implementations of AR-PAM to our high-resolution implementation of OAT with SPADE, where typical values of various implementations are cited.

**Characteristic performance criteria of piezo-based AR-PAM, RSOM, and SPADE**			
	Conventional AR-PAM	RSOM	SPADE-based OAT
Central frequency (MHZ)	50[5]	100[7,8]	75 -115*
Bandwidth (MHZ)	35-50[5,9]	160-190[7,8]	>200 *
Detector dimensions	Diameter: 6.2 mm	Diameter: 1.5-3.6 mm[10]	0.5×30-200 µm
Axial-spread-function width (µm)	15-27[9,11]	4[7,10]	~ 6-13*
lateral-spread-function width (µm)	47-87[9,11]	~20[7,10]	< 20
Focal distance (mm)	6[9]	1.3-2[7,8,12]	-
Depth of field (mm)	~0.29[13]	0.046-0.11[13]	-
NEP (mPa/√Hz)	0.2[12]	1.1[12]	2.2-9.8

Supplementary Table 1| A comparison between the characteristic performance criteria of piezo-based AR-PAM, RSOM, and SPADE. (*) the two values for the central frequency, bandwidth, and axial spread function relate to the raw measurement and the calibrated measurement in which the limited response of the photodetector was corrected.

We note that the exact values summarized in Supplementary Table 1 are specific to the design parameters and manufacturing quality of each transducer and may also be affected by the method used to measure these values. Nonetheless, the clear distinction between conventional AR-PAM and RSOM is clear, and is mostly dictated by the different central frequencies and bandwidth employed, as dictated by

Supplementary Eqs. 1a,b. The values for the depth of field were calculated from the experimental transducer parameters using the analysis of Rosenthal et al.[13]. The NEP values are taken from Winkler[12] and represent the fundamental sensitivity limit dictated by thermal noise, rather than a measured value. It is important to note that the NEP for focused transducers represents the sensitivity to a very small volume, determined by the lateral-spread-function width and the depth of field; in contrast, the NEP values of SPADE are representative for any source location. Thus, although the transducer sensitivities achievable by RSOM and SPADE are comparable, the measured acoustic signal in SPADE-based OAT, received from a larger volume, is expected to be much higher when a direct comparison is performed.

Supplementary Note 3: Comparison to optical detectors of ultrasound

Supplementary Table 2 compares the performance of SPADE to competing optical platforms for ultrasound detection. So far, only polymer Fabry-Pérots have been demonstrated *in vivo* in a tomographic configuration, but their dimensions and bandwidth are comparable with those of piezoelectric transducers used in OAT. Polymer micro-rings achieve considerably larger bandwidths and sensitivity than planar Fabry-Pérot resonators for the same diameter, but have only been demonstrated for OAT of point sources. Silicon-photonics detectors in which the detection in performed on the facet have achieved an exceptional combination of size, bandwidth, and sensitivity, but their application for high-fidelity OAT has been limited by parasitic effects. Finally, the use of membranes to increase the sensitivity of silicon-photonics detectors comes at the price of reduced bandwidth, limiting the achievable resolution.

**Optical detectors of ultrasound**					
		Dimensions (µm, H×W×L)	Bandwidth (MHz)	NEP (mPa/√Hz)	*In vivo* demonstration in OAT
Polymer Fabry-Perot	planar	22-38×64×64[4]	22[4]-55[14]	47-69[4]	Yes
	Plano-concave[15]	340×12.5×12.5<br>16 ×12.5×12.5	2.8<br>20	1.6<br>2.1	No
Polymer micro-ring[16]		1.4×60×60	350	5.61	No
Silicon-photonics: facet detector[1]		0.2×0.2×0.2	230	9	No
Silicon-photonics: membrane detector[17]		0.2×15-20×15-20	27	1.3-2	No
Silicon-photonics: SPADE		0.2×0.5×30-200	> 200	2.2-9.8	Yes

Supplementary Table 2| Comparison of different optical detectors of ultrasound.

Supplementary Method 1: Theoretical sinogram.

The leading effects observed in the sinograms of Fig. 4b may be readily produced in numerical simulations that model the propagation of longitudinal waves, which propagate from the source to the chip in the aqueous medium at a speed of v_l, and surface acoustic waves (SAWs) which propagate on the chip's surface at a speed of v_s. When the propagation is only of longitudinal waves, the time it takes the signal

to be detected is determined by the time-of-flight principle and is equal to $T = L/v_l$, where L is the distance of the source from the detector. When the source is scanned over the x axis (Supplementary Fig. 1a), one obtains $T = \sqrt{h^2 + x^2}/v_l$, where h is the axial distance (z axis) between the source and the SPADE, i.e. the relation between T and x is hyperbolic. In the case of SAW generation, the acoustic propagation involves two parts: a longitudinal wave that propagates towards the chip at an angle θ and a SAW that propagates the rest of the distance on the surface of the chip. Since a SAWs can be created only at a single value of θ [18], one may assume that θ is constant when the source is scanned over the x axis. In that case, one obtains $T = (x - h\cot\theta)v_s^{-1} + hcsc\theta v_l^{-1}$, i.e. a linear relation between T and x. Supplementary Fig. 1b and 1c respectively show the simulated sinograms for the experimental parameters of the measurements of Fig. 4b for the time-of-flight and SAW models, whereas Supplementary Fig. 1d shows a sum of the two sinograms. The SAW in Supplementary Fig. 1d reaches the detector at shorter times, since the propagation velocity of the SAW (v_s) in the SPADE's substrate is substantially faster than the propagation velocity of the longitudinal waves in water (v_l).

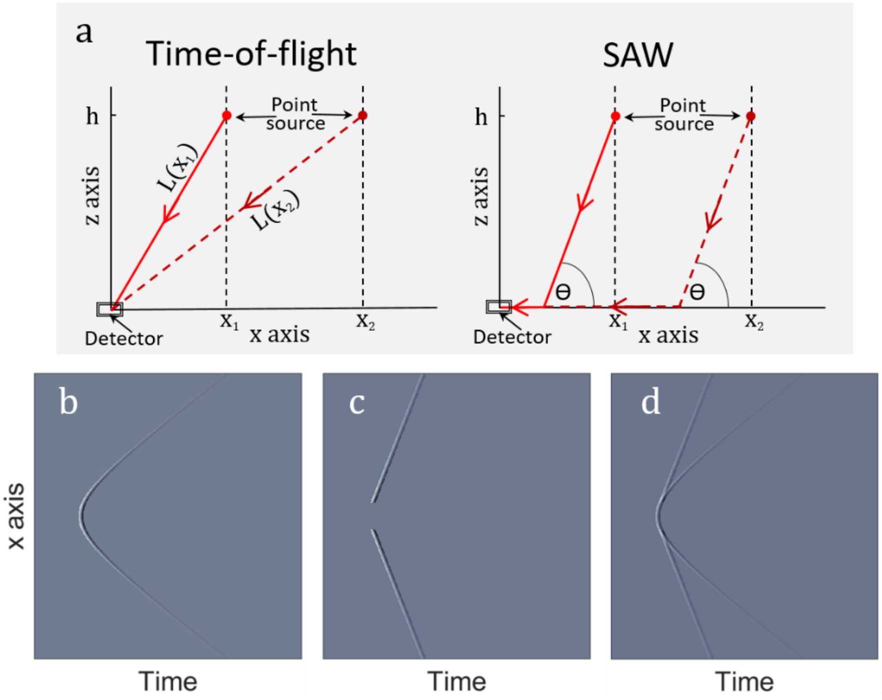

Supplementary Fig. 1| Theoretical sinogram. a, Acoustic propagation trajectories for longitudinal waves (time-of-flight) and SAW. **b,** Simulated sinogram of a longitudinal wave governed by the time-of-flight principle. **c,** Simulated sinogram of a SAW. **d,** Simulated sinogram including both a longitudinal wave and SAW.

Supplementary Method 2: SPADE with the TM mode.

One way to further increase the sensitivity of the SPADE is to work with transvers magnetic (TM) waveguides, rather than transvers electric (TE) waveguides. The sensitivity enhancement in TM waveguides results from two reasons: lower propagation loss enabling the fabrication of higher Q-factor resonators and the mode cross-section that penetrates deeper into the PDMS over-cladding (Supplementary Fig. 2g,h). As seen in Supplementary Fig. 2, the peak-to-peak signals acquired by the TM sensor (Supplementary Fig. 2e,f) is 2.4 times greater than the peak-to-peak signals acquired by the TE

sensor (Supplementary Fig. 2a,b). Furthermore, the relative suppression of the SAW (marked in red arrows in Supplementary Fig. 2) in the TM sensor is clearly seen in Supplementary Fig. 2f. This TM sensor was not used for imaging purposes in this work since its detection area is highly elongated along the waveguide (approximately 200 µm), making it unsuitable for high resolution imaging purposes. The light localization and hence the effective detection area, can be more localized by design and manufacture of higher Q-factor resonators.

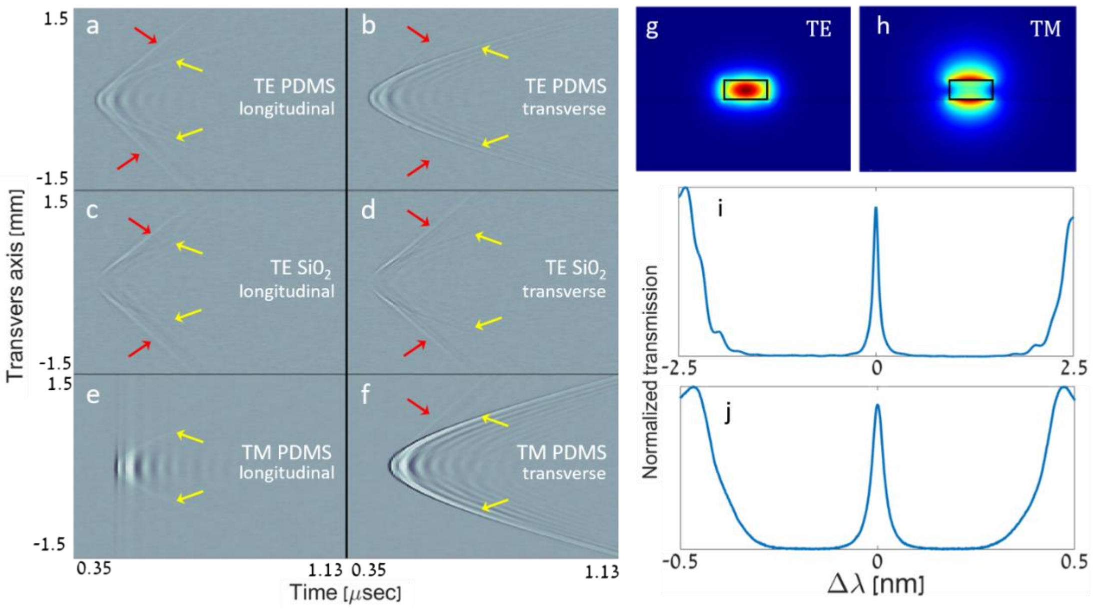

Supplementary Fig. 2| Comparison of TE and TM waveguides. Sinogram signals acquired with: **a,** TE resonator with PDMS over-cladding scanned parallel to the bonded fibers; **b,** TE resonator with PDMS over-cladding scanned perpendicular to the bonded fibers; **c,** TE resonator with a silica over-cladding scanned parallel to the bonded fibers; **d,** TE resonator with a silica over-cladding scanned perpendicular to the bonded fibers; **e,** TM resonator with PDMS over-cladding scanned parallel to the bonded fibers; **f,** TM resonator with PDMS over-cladding scanned perpendicular to the bonded fibers. In **a-f,** red arrows mark surface acoustic waves, yellow arrows mark direct-impact acoustic waves. **g,** Simulated TE-mode distribution in the waveguide cross-section. **h,** Simulated TM mode distribution in waveguide cross-section. **i,** Normalized transmission spectrum of the TE resonator. **j,** Normalized transmission spectrum of the TM resonator.

Supplementary Method 3: SPADE bandwidth measurement.

The acoustic signal measured for a point-like optoacoustic source (Fig. 4h) represents the inherent response of the π-BG resonator to acoustic waves, i.e. the conversion from pressure to an optical signal, convolved with the source waveform and electro-optic response of the photodiode. Accordingly, to assess the inherent bandwidth of SPADE, corresponding only to its optical and acoustic components, its measured spectrum needs to be divided by the optoacoustic source spectrum and electro-optic spectral response of the photodiode. While ideally one would measure both these spectra, a calibrated measurement of a weak acoustic source over bandwidths exceeding 200 MHz poses numerous experimental challenges. Therefore, we used the theoretical temporal response for an optoacoustic point source, a derivative of a delta function, corresponding to a spectral response which is a linear function of the acoustic frequency.

The spectral response of the photodiode can be measured by illuminating it with an optical beam whose intensity varies rapidly over time, e.g. a short optical pulse or a white-noise source. In this work, a noise-source was employed using the same procedure described by Volodarsky et al.[19]. Briefly, the optical white-noise source was an erbium-doped fiber amplifier, generating amplified spontaneous emission optically filtered to a bandwidth of 4 pm by a π-BG resonator. An analysis of the noise characteristics of this source may be found in Rosenthal et al.[20]. The theoretical acoustic spectrum of the source, the measured electro-optic spectrum of the photodiode, and the combined calibration spectrum are shown in Supplementary Fig. 3. We note that since the optoacoustic source is not necessarily ideal, and may include diffraction effects from the fiber tip, the resulting calibration spectrum is merely an approximation, which is useful for bandwidth estimation, but not for quantitative signal correction and image reconstruction.

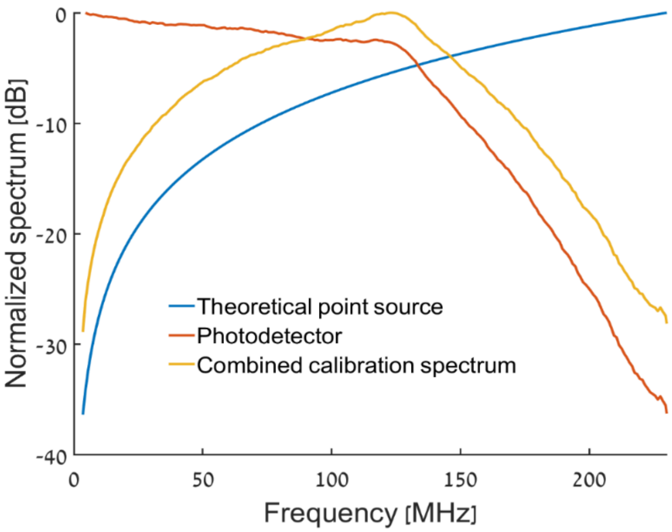

Supplementary Fig. 3| SPADE bandwidth measurement. The theoretical acoustic spectrum of an optoacoustic point source (blue), the measured electro-optic spectrum of the photodiode (red), and the combined calibration spectrum (yellow).

Supplementary Method 4: 3D tomographic reconstruction from SPADE.

The mouse ears presented in Fig. 5 are the maximum intensity projection of the 3D tomographic reconstruction. Here we present six tomographic slices of the three-dimensional information captured from the mouse ear (Fig. 5d) as a montage in Supplementary Fig. 4. These six tomographic slices show the three-dimensional information captured, presented at depth difference of 30 µm between each consecutive slice. The depth information along with the spatial resolution is clearly visible in Supplementary Fig. 4, emphasizing the microscopic resolution achieved by far-field tomography using SPADE. Furthermore, Supplementary Fig. 5 presents the raw opto-acoustic signals measured in a single line-scan, showing multiple hyperbolic signals, typical with tomographic measurements of complex object.

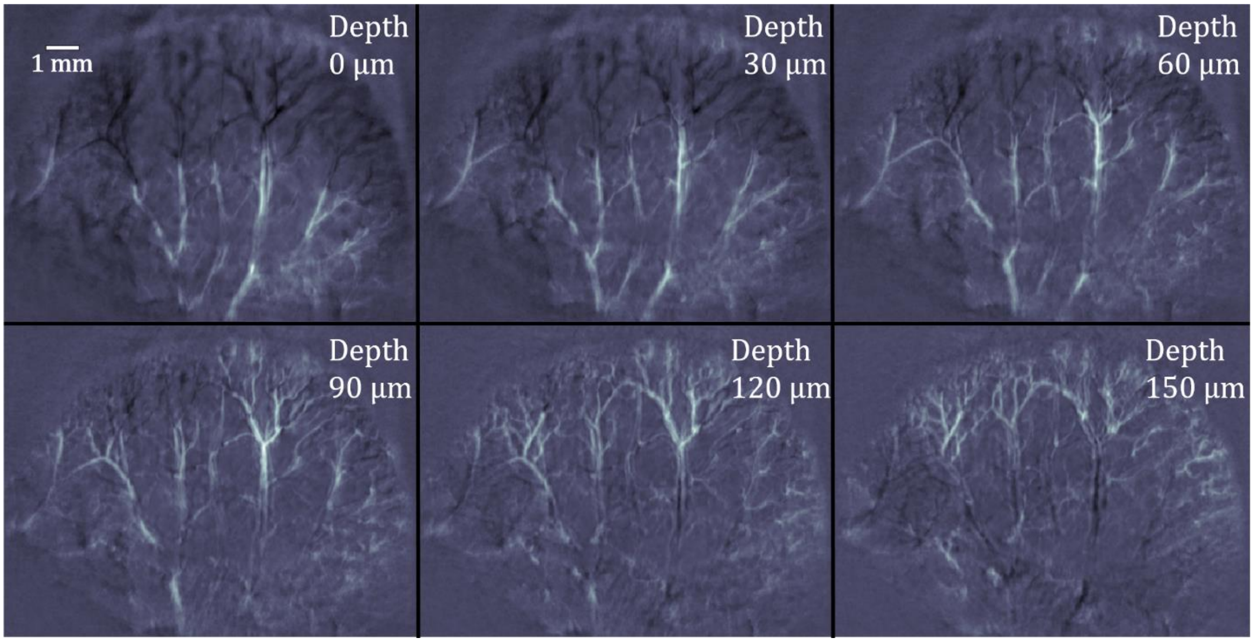

Supplementary Fig. 4| Montage of different tomographic depth of mouse ear imaging. Six tomographic slices of mouse ear presented in Fig. 5d. The depth difference between each consecutive slice is 30 µm.

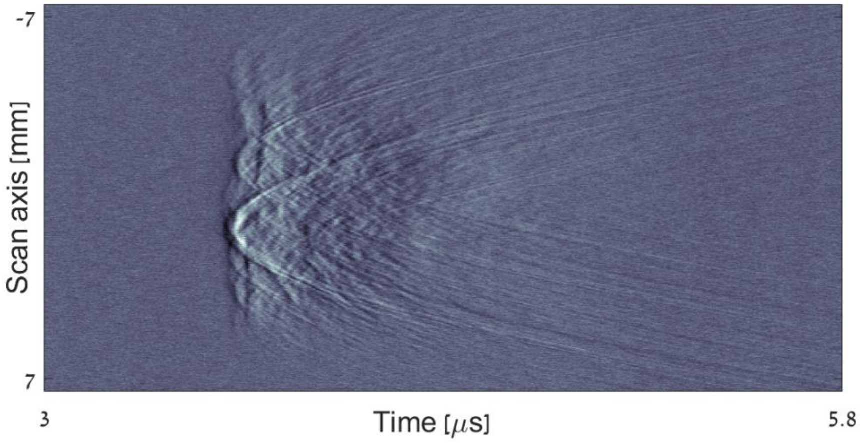

Supplementary Fig. 5| Raw signal sinogram from mouse ear opto-acoustic imaging. Optoacoustic signals from a single line-scan of mouse ear presented in Fig. 5d and Supplementary Fig. 4, showing typical tomographic hyperbolic signals.

Supplementary References

1. Shnaiderman, R. *et al.* A submicrometre silicon-on-insulator resonator for ultrasound detection. *Nature* **585**, 372–378 (2020).
2. Xu, M. & Wang, L. V. Analytic explanation of spatial resolution related to bandwidth and detector aperture size in thermoacoustic or photoacoustic reconstruction. *Phys. Rev. E* **67**, 056605 (2003).
3. Rosenthal, A., Razansky, D. & Ntziachristos, V. High-sensitivity compact ultrasonic detector based on a pi-phase-shifted fiber Bragg grating. *Opt. Lett.* **36**, 1833–1835 (2011).
4. Zhang, E., Laufer, J. & Beard, P. Backward-mode multiwavelength photoacoustic scanner using a planar Fabry-Perot polymer film ultrasound sensor for high-resolution three-dimensional imaging of biological tissues. *Appl. Opt., AO* **47**, 561–577 (2008).
5. Moothanchery, M. & Pramanik, M. Performance Characterization of a Switchable Acoustic Resolution and Optical Resolution Photoacoustic Microscopy System. *Sensors* **17**, 357 (2017).
6. Hindelang, B. *et al.* Non-invasive imaging in dermatology and the unique potential of raster-scan optoacoustic mesoscopy. *Journal of the European Academy of Dermatology and Venereology* **33**, 1051–1061 (2019).
7. Omar, M., Gateau, J. & Ntziachristos, V. Raster-scan optoacoustic mesoscopy in the 25–125 MHz range. *Opt. Lett., OL* **38**, 2472–2474 (2013).
8. Omar, M., Aguirre, J. & Ntziachristos, V. Optoacoustic mesoscopy for biomedicine. *Nat Biomed Eng* **3**, 354–370 (2019).
9. Li, M.-L., Zhang, H. F., Maslov, K., Stoica, G. & Wang, L. V. Improved in vivo photoacoustic microscopy based on a virtual-detector concept. *Opt. Lett., OL* **31**, 474–476 (2006).
10. Aguirre, J. *et al.* Broadband mesoscopic optoacoustic tomography reveals skin layers. *Opt. Lett., OL* **39**, 6297–6300 (2014).
11. Moothanchery, M., Dev, K., Balasundaram, G., Bi, R. & Olivo, M. Acoustic resolution photoacoustic microscopy based on microelectromechanical systems scanner. *Journal of Biophotonics* **13**, e201960127 (2020).
12. Winkler, A. M., Maslov, K. I. & Wang, L. V. Noise-equivalent sensitivity of photoacoustics. *JBO* **18**, 097003 (2013).
13. Rosenthal, A., Ntziachristos, V. & Razansky, D. Acoustic Inversion in Optoacoustic Tomography: A Review. *Curr. Med. Imaging Rev.* **9**, 318–336 (2013).
14. Baumann, E. *et al.* A backward-mode optical-resolution photoacoustic microscope for 3D imaging using a planar Fabry-Pérot sensor. *Photoacoustics* **24**, 100293 (2021).
15. Guggenheim, J. A. *et al.* Ultrasensitive plano-concave optical microresonators for ultrasound sensing. *Nat. Photon.* **11**, 714 (2017).
16. Zhang, C., Chen, S.-L., Ling, T. & Guo, L. J. Review of Imprinted Polymer Microrings as Ultrasound Detectors: Design, Fabrication, and Characterization. *IEEE Sensors Journal* **15**, 3241–3248 (2015).
17. Westerveld, W. J. *et al.* Sensitive, small, broadband and scalable optomechanical ultrasound sensor in silicon photonics. *Nature Photonics* 1–5 (2021) doi:10.1038/s41566-021-00776-0.
18. Rosenthal, A. *et al.* Embedded ultrasound sensor in a silicon-on-insulator photonic platform. *Appl. Phys. Lett.* **104**, 021116 (2014).
19. Volodarsky, O., Hazan, Y. & Rosenthal, A. Ultrasound detection via low-noise pulse interferometry using a free-space Fabry-Perot. *Opt. Express* **26**, 22405–22418 (2018).
20. Rosenthal, A., Razansky, D. & Ntziachristos, V. Wideband optical sensing using pulse interferometry. *Opt. Express* **20**, 19016 (2012).
